# Benchmarking the university campus food environment and exploring student perspectives about food insecurity and healthy eating: a case study from Australia

**DOI:** 10.1186/s12889-024-18664-x

**Published:** 2024-05-06

**Authors:** Jemma Keat, Putu Novi Arfirsta Dharmayani, Seema Mihrshahi

**Affiliations:** Department of Health Sciences, Faculty of Medicine, Health and Human Sciences, Macquarie University, Level 3, 75 Talavera Road, North Ryde, Sydney, NSW 2109 Australia

## Abstract

**Objective:**

To benchmark the university food environment and explore students’ experiences with food insecurity and healthy eating in order to inform interventions to improve access and affordability of healthy foods for university students.

**Design:**

A food environment audit was conducted on the university campus using the Uni-Food tool from April to May 2022 and was comprised of three main components, university systems and governance, campus facilities and environment, and food retail outlets. A qualitative study design was also used to conduct focus groups and semi-structured interviews with students to explore key themes regarding their experiences with food insecurity and healthy eating.

**Setting:**

Macquarie University, Australia.

**Participants:**

For the food environment audit 24 retail outlets on campus and for the qualitative component 29 domestic and international students enrolled at Macquarie University.

**Results:**

The university only scored 27% in total for all components in the food environment audit. The results showed the need for better governance and leadership of the food environment. The qualitative component suggested that the main barriers to accessing healthy foods were related to availability, pricing, and knowledge of healthy foods. Future intervention ideas included free fruits and vegetables, food relief, discounts, improved self-catering facilities, education, and increased healthy food outlets.

**Conclusions:**

Improving governance measures related to healthy eating on campus are a core priority to strengthen the food environment and students identified pricing and availability as key issues. These findings will inform effective and feasible interventions to improve food security and healthy eating on campus.

**Supplementary Information:**

The online version contains supplementary material available at 10.1186/s12889-024-18664-x.

Food environments are a collective of physical, socio-cultural, economic, political factors that influence the availability, accessibility, and consumption of foods and beverages [1]. Food environments can act as a facilitator or barrier to health, depending on the presence of ultra-processed, energy-dense foods and fresh food consumption [1]. The components of food environments include availability, accessibility, promotion and marketing, affordability, quality, convenience and governance[1, 2]. Due to their influential nature, food environments can influence experiences with food, leading to food insecurity [1].

Food insecurity occurs when individuals or communities lack secure access to sufficient amounts of safe, nutritious food for an active, heathy life [1]. Additionally, the scarcity of safe foods can contribute to poorer mental and social wellbeing for these individuals[2, 3]. Food insecurity can be influenced by a multitude of complex barriers and enablers, including an individual’s food environment. Poor food environments which can exacerbate food insecurity and unhealthy dietary behaviours include those that have limited access to healthy, affordable food outlets due to unavailability or location, and the increased presence and promotion of unhealthy, affordable fast-food outlets which incentivise poor dietary practices [4].

Young people are more at risk of food insecurity due to the additional constraints that they are faced with during this transitional period including, sociocultural influences, living out of home for the first time and targeted marketing [5]. Additionally, young people may be susceptible to consuming unhealthy foods or skipping meals due to low availability and accessibility of healthy, fresh, and culturally appropriate foods on university campuses [6]. University food environments are complex and highly influential in shaping students’ dietary behaviours and experiences with food security [6]. In Australia, the prevalence of food insecurity had been estimated to be up to 15% prior to the COVID-19 pandemic, and our research has estimated the prevalence of food insecurity among university students during the pandemic in 2020 to be 42%, and up to 75% in international students [7].

Universities often have a variety of food outlets on campus however, these may be inequitable for students due to heightened pricing and low fresh food availability [6]. Over the past decade, many universities in Australia have shifted away from internally managed university cafeterias to commercial models within their food environments [6]. As these models are predominately economically driven, this increases the risk of inequities experienced by students in regard to healthy, affordable food options on campus. As students spend most of their time on campus, it is essential to provide a supportive food environment that enables and promotes the consumption of sufficient safe and nutritious food to adopt a healthy lifestyle whilst considering their socio-economic status [6]. Therefore, there is a need to assess the food environment and students’ experiences with university food environment to address food insecurity among university students.

To date, a number of studies have focused on student’s perspectives on food choices on campus, but not specifically examined their experiences with food insecurity in relation to the University food environment[8–10]. Thus, this research aims to benchmark the food environment at an Australian university using a validated comprehensive tool [11] and assess students’ experiences with the university food environment including their experiences with food insecurity and healthy eating. The ultimate goal of the research is to inform interventions to reduce food insecurity on campus and improve access and affordability of healthy foods for university students.

## Methods

### Setting

This study was conducted at Macquarie University, located in Sydney, Australia. The university is a public institution which has more than 44,000 domestic and international students enrolled across 100 countries. There are four main faculties across the university including then disciplines of arts, business, medicine, health and human sciences, and science and engineering.

### Food environment benchmarking

#### Design and sampling

The university food environment was assessed utilising the Uni-Food tool developed by Deakin University [11]. This tool was selected as it encompassed a multi-faceted approach which includes various elements of the educational food environment, rather than focusing on a singular component as evident in other assessment tools [11]. Due to the complex nature of university food environments, it is crucial to conduct a comprehensive assessment of this setting to inform future interventions [11].

The Uni-Food tool focused on the measurement of three main components of the university food environment including, university policies and governance systems, food retail outlets and campus facilities and environment [11]. There are a total of 68 indicators within the tool, across these components [11]. Within each section of the tool, there are 16 domains and 42 sub-domains which provide a comprehensive analysis of the university environment [11]. The retail outlets component of the audit was conducted in May 2022 and involved on-campus observations and brief discussions with retail staff of 24 food outlets on campus.

#### Data collection

**Fig. 1:**
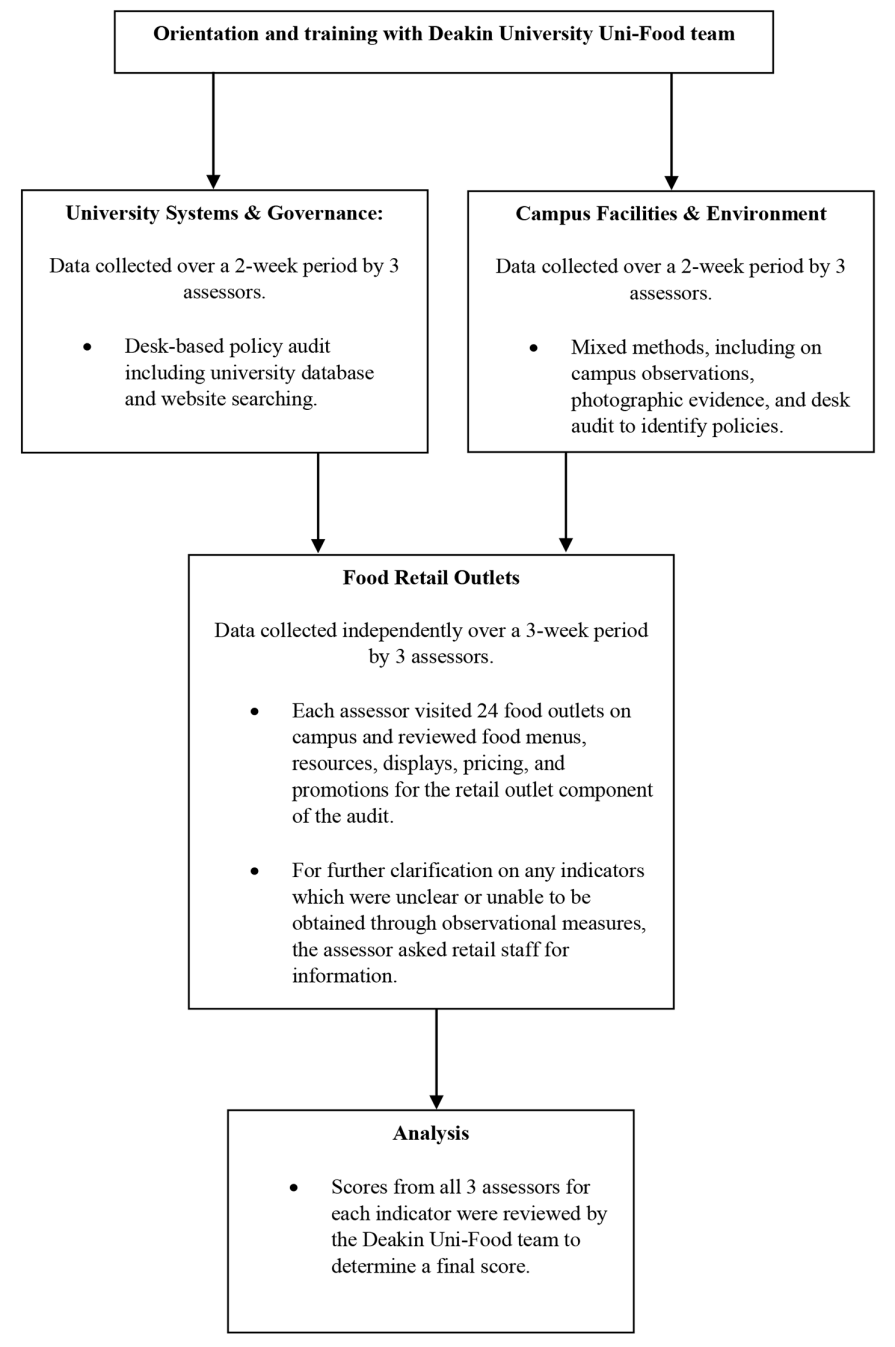
Uni Food Tool Data Collection Process

A comprehensive benchmarking of the university food environment was conducted in April to May 2022 by three assessors from Macquarie University using the Uni-Food tool [11]. Data was collected based on policy audit, campus audit, assessment of campus food environments using the Uni-Food Tool by three assessors after completing a training session. Figure 1 depicts the process for implementing the University Food Environment Assessment (Uni-Food) tool.

### Students focus group/interviews

#### Design and recruitment

Shortly after the Food Environment Benchmarking was completed, a qualitative study was conducted using focus groups and semi-structured interviews with students. The inclusion criteria to be participants were aged 18 years or older, enrolled as a student at Macquarie University, and identified as ‘food insecure’ using Six-Item Short Form Food Security Survey Module [12]. Demographic data was also collected as part of the pre-screening survey which identified age, enrolment status, student status (domestic or international), and race/ethnicity.

Recruitment of students to took place from August to September 2022. Participants were recruited using various methods, including university communications, social media advertisements, and student associations. Participants were asked to provide informed consent to participate in the study using a Digital Patient Information and Consent Form (PICF). The research team contacted them via their nominated student email as provided in the contact details section of this form.

#### Data collection

Focus groups and interviews were conducted between 12th August 2022 and 9th September 2022 either online or in person. Focus group and interview sessions lasted between 45 and 60 min and were digitally recorded using Microsoft Teams. Each focus group session consisted of three to five participants. Domestic and international students were allocated to separate sessions to ensure that the conditions were supportive of their enrolment status and potential language barriers. Open ended questions were asked during these sessions to allow students to discuss their experiences and ideas in regard to food insecurity and healthy eating on campus (Supplementary Material S1).

### Data analysis

#### Food environment analysis

For all components of the Uni-Food tool, scores from all three assessors were then reviewed by the Uni-Food team at Deakin University who determined a final score, this was to improve the reliability of the results and reduce reporting biases. If any major discrepancies were identified amongst assessors, the reviewer then used the evidence supplied to make a final decision on the score.

#### Student focus group/interviews

Qualitative data from the focus groups and interviews was analysed using the NVivo 12 analytical software. A reflexive thematic analysis approach was undertaken as this method is effective in understanding experiences with increased flexibility in comparison to other methods which follow specific theoretical frameworks. The six steps of reflexive thematic analysis were utilised to analyse the datasets, including data familiarisation, coding, themes, and review of themes, and definitions [13].

The transcriptions were checked for accuracy against the audio recordings. The transcripts were then coded after they were initially reviewed to identify key themes and ideas from students in relation to various concepts discussed during the sessions. Codes were allocated to highlight students’ feelings and experiences with food insecurity and healthy eating on campus. These were then further refined through a secondary review of transcripts and coding.

Following this, the codes were reviewed and grouped together based on the broader concept of each code to create a wider theme as emerged throughout the sessions. After refinement of the themes, a further analysis of each theme was conducted to understand the deeper meaning and ideas shared by students. A total of 8 themes were included in the final analysis, with a total of 30 sub-themes as outlined in Supplemental Figure S2.

## Results

### Food environment analysis

According to the Uni-Food analysis, Macquarie University scored 27 out of 100 (27%) in total. Food retail outlets scored 45 out of 100 (45%), campus facilities and environment scored 41 out of 100 (41%), and university systems and governance only scored 4 out of 100 (4%). These results are concerning and highlight the significant gaps within each component of the university food environment, particularly within the university’s governance system as the lowest scoring component.

#### University systems and governance

**Fig. 2:**
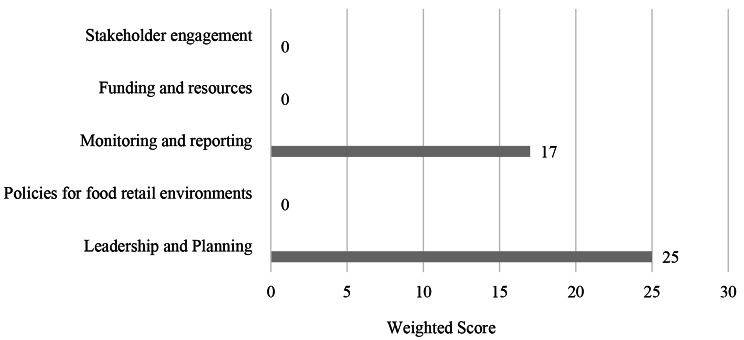
University systems and governance results

This component scored 4 out of 100 (4%), which was the lowest scoring component overall and held a weighting of 40%. The university has implemented sufficient sustainability frameworks however, these did not have a focus on food, this is highlighted within leadership and planning (25%). Additionally, there were no university-wide policies that aimed to govern the procurement of healthy foods and beverages on campus. Stakeholder engagement was absent and there were no strong partnerships relative to healthy eating on campus. These results are shown in Fig. 2.

#### Campus facilities and environment

**Fig. 3:**
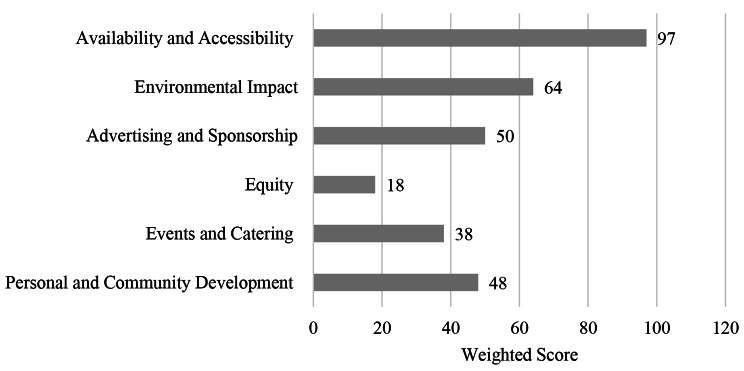
Campus facilities and environment results

This component scored 41 out of 100 (41%) and held a weighting of 40% as outlined in Fig. 3. The highest scoring domains was availability and accessibility (97%), whereas the lowest scoring domains was equity (18%). Whilst this score highlights the needs for improvements, some key strengths were identified in this component, such as the availability of culturally diverse food on campus, free drinking water widely available across campus, the availability of self-catering facilities at some dedicated location on campus. Moreover, the university also has strong waste management practices in place, including separated bins and sorting processes to ensure recycling occurs, as well as adequate personal and community development initiatives, including a community garden for students and the community. There were also evident educational initiatives regarding sustainability and healthy diets however, these were driven by student groups with limited involvement from the university.

#### Food retail outlets

**Fig. 4:**
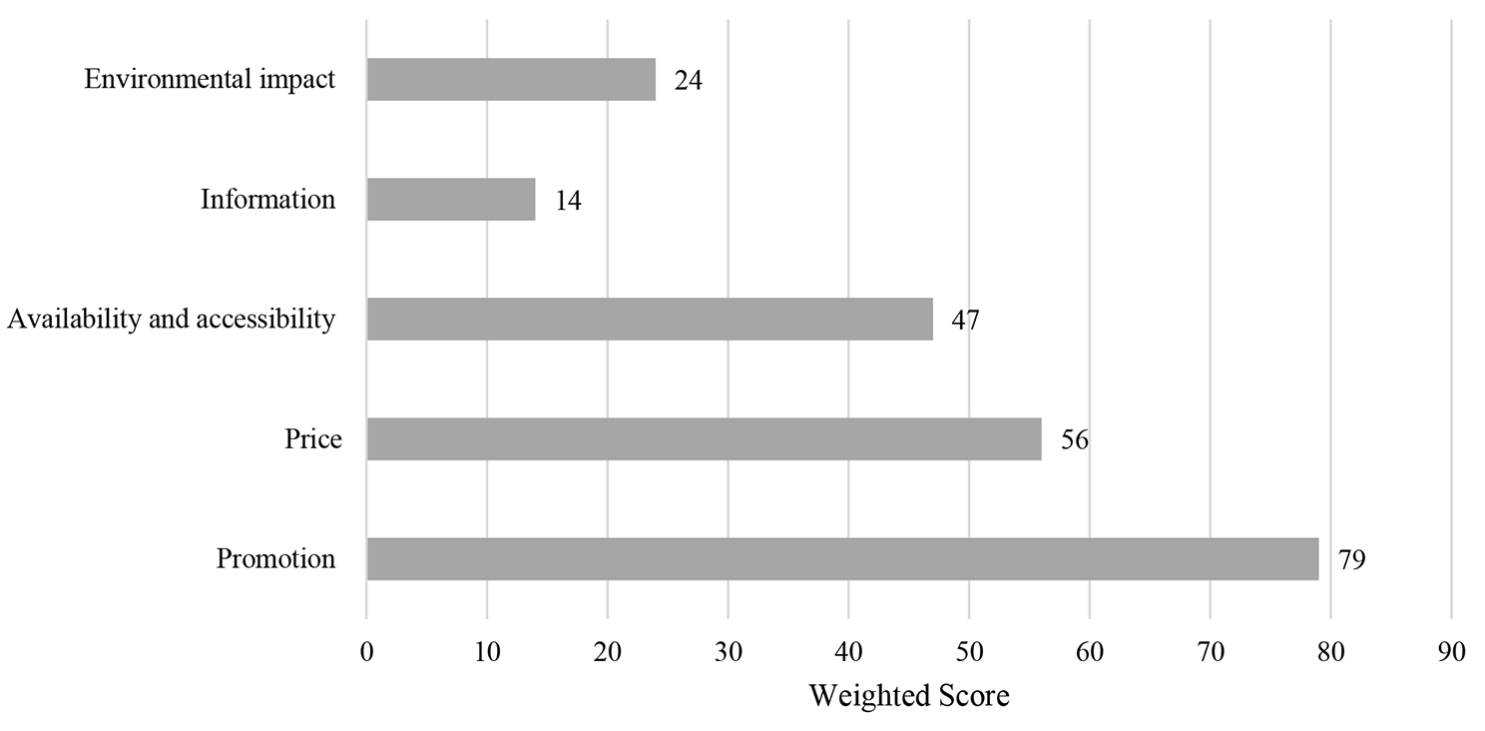
Food retail outlet results

This component was the strongest of all components within the audit and scored 45 out of 100 (45%), with a weighting of 20%, highlighted in Fig. 4. The highest scoring domains was promotions (79%), whereas the lowest scoring was information (14%). Promotions were a key strength as 15 out of the 24 food outlets (62.5%) were free from any promotions of unhealthy foods and beverages. In majority of the food outlets on campus, prices equally encourage the purchase of unhealthy and unhealthy foods as well as vegetarian and meat-containing options. Whilst there is no strong incentive to opt for the purchase of healthy and vegetarian foods, there is no significant encouragement to purchase unhealthy options which can be considered a strength.

Approximately 10 of the 24 food outlets sold predominantly unhealthy foods or beverages, with the remaining 14 offering either majority healthy options or a combination of both healthy and unhealthy food options. Most food outlets lacked sufficient nutritional information and only 14 of the 24 outlets provided very limited information on dietary requirements and common allergens. A significant gap was highlighted within the waste management domain as whilst many outlets utilised recyclable or reusable packaging, no food outlets had established waste monitoring and strong reduction systems.

### Student focus groups and interviews

#### Demographic characteristics

A total of 48 participants completed the online screening survey and provided informed consent. After following up with the participants who had initially registered to participate, a total of 29 students were included in the analysis, with 17 (58.6%) were postgraduate students, and 12 (41.4%) were undergraduate. There were 13 (44.8%) domestic students and 16 (55.2%) international students with age range between 18 and 36. Participants’ ethnicities included Asian (34.5%), Australian (17.2%), Indian (10.3%), Indigenous (10.3%), and others including Arabic, British, Scandinavian and Sri Lankan (22.7%).

Results from the qualitative component of the study were grouped into themes (1) Healthy food options on campus (2), Influences of food choices on campus (3), Changes in eating habits since starting at university (4), Impacts of COVID-19 on eating habits (5), Health impacts of food insecurity (6), Barriers to accessing healthy food (7), Views on current university support, and (8) Future intervention ideas. Representative quotes for each theme and sub-theme are shown in Table 1.

**Table 1:** Theme and sub-themes associated with campus food environment, eating habits, and food insecurity (*n* 29)

Theme	Sub-theme	Illustrative quotes	Code
**Healthy food options on campus**	Fruits & vegetables	*“I would say probably the healthiest options that come to mind for me would be (…) because they have salads, or it would be the juice place”.* *“Any of the options that have like salads of heaps of veggies”.*	*P203, Domestic* *P203, Domestic*
	Food safety	*“Umm unclean food. You know what came to my mind when food is not clean, you know, for example, it will contain some bacteria”.* *“I have a Chinese background, so I tend to eat some Asian food, but then one day I found plastics and the other day I found hairs in it”.*	*P55, Domestic* *P161, International*
	Nutritional balance	*“I will say it’s very limited about the healthy food on campus and I don’t feel there’s one that’s very healthy. if I want to eat healthy, it’s probably just portion control from myself”.*	*P60, International*
	Sandwiches	*“Sandwiches and the other stuff are healthy and yeah, I think, yeah, it’s my only choice on the campus”.*	*P68, International*
**Influences of food choices on campus**	Social & cultural influences	*“If people in a group have unhealthy food, we tend to have unhealthy food with them”.* *“…not finding like any Arabic food that is related to my culture. It would mean like, no, I’m not going to buy anything. Just stick with the one that I know, so I may like to go to a shopping centre to buy things that I already know and cook it, take it with me to the uni if I have time”.*	*P66, International* *P68, International*
	Nutrition	*“The kind of food that I eat that will give me more vitamins that will give me more energy to sustain my daily activities”.* *“Do they have any vegetables in it? Like does it provide me enough energy to like to help me sustain during the whole study periods”.*	*P55, Domestic* *P167, International*
	Convenience	*“If I can’t grab it very quickly, I probably won’t get it, and if it’s more expensive than like an unhealthy option, that’s fast, I wouldn’t get it”.* *“Primarily would be prices, yeah, and accessibility like how close they are to campus”.*	*P130, International* *P114, International*
	*Price*	*“Like trying to get the cheapest but also the healthiest, but there aren’t that many that are like cheap and healthy, so it feels choosing between the two, definitely cheapness”.* *“There are also shops or they have restaurants in the campus which provide healthy food, but they’re too overpriced, so people tend to have like a cheaper option, like, unhealthy food”*	*P203, Domestic* *P66, International*
	*Taste preferences*	*“Actually, I would prefer like it has to be delicious instead of the price, yeah”.* *“Price and Taste cause technically the new toasty place that opened is pretty cheap. I got a toast sandwich for $4.90 umm, which was great, but it didn’t taste very nice”.*	*P167, International* *P210, Domestic*
***Changes in eating habits since starting at university***	*Time*	*“I don’t have time between classes so like even today like 5 days for example, I have classes that go from 9:00 AM to 4:00 PM back-to-back so even if I did want to eat, I don’t have time to eat”.*	*P204, Domestic*
	*Energy*	*“Things that are really quick that I can eat on the go and a lot of those things aren’t necessarily healthy and so that kind of or gives me a lot of energy in the long run, so I find myself a bit more tired, a bit more sluggish”.*	*P84, Domestic*
*Impact of COVID-19 on eating habits*	*Access to shops*	*“Sometimes less like when you when you couldn’t go out of your like radius, I guess I was restricted to like the food in my own area I guess and so, in that sense, had less control over what I could eat”.*	*P118, Domestic*
	*Reduced stock*	*“With COVID there was like a bit of access in terms of there was I think like what was even on the shelves was a lot less than normal, so it was like less options I’d say umm junk food was definitely this like this a simple easy option”.*	*P210, Domestic*
	*Reduced consumption*	*“I like eating three times a day like, but I skip to let me say twice a day, but I like snacks in my life but there was not enough money for me to buy the snacks”*	*P58, International*
	*Pricing*	*“Obviously the price is for fresh fruit and vegetables are a lot pricier than they used to be. One thing that my family and I enjoy is, you know, frozen vegetables and you just heat them up. I think that they’re a great alternative, and they taste just as good as I think, in my opinion”.*	*P85, Domestic*
	*Health consciousness*	*“The pandemic has actually changed for the better. Uh, you know how everyone in my family actually cooking a lot more food at home and instead of buying of from of food outside”.*	*P65, International*
***Health impacts of food insecurity***	*Physiological changes*	*“I was experiencing issues like with the weight gain, but also like there’s this brief period where like I was having a lot of hair loss, which was really concerning, and I found myself having like no energy”.*	*P130, International*
	*Mental health*	*“Definitely stressed out, say, because I would like to eat healthy and then it makes me very stressed about like body image and I guess not being able to access healthier foods”.* *“It definitely would impact my mental health, especially if I’m not consuming like enough nutritious kind of foods and would also affect my weight as well”.*	*P203, Domestic* *P190, Domestic*
***Barriers to accessing healthy foods***	*Pricing*	*“The one thing that prevents me eating healthy food is back to price related because, I feel like vegetables in Australia because inflation or something, it’s more expensive than meat, like it’s not logical in my mind”.*	*P60, International*
	*Availability*	*“One of the girls in my classes is vegan and we’ll go out and we’ll try and get something for lunch or something to eat after class and she can’t have any of the sushi, it’s more complicated than you’d expect”.*	*P210, Domestic*
	*Knowledge*	*“I feel like a lot of people tend to think vegetables, they must be healthy, that’s it, you know, but I think like an educational barrier, I guess, like having a more holistic understanding of what healthy is, it’s not just eating veggies all the time and like more education like in nutritional stuff I guess”.*	*P142, International*
***Views on current university support***	*University education*	*“The gym had sent like a nutrition class thing I got an e-mail that they’re doing something for information of nutrition for building muscle and also one about eating disorders”.*	*P210, Domestic*
	*Food relief boxes*	*“No healthy eating like they were all canned food and all those things. But they used to provide like dairy products, milk, and normal basic stuffs but they were not too healthy, but they were like sufficient”.* *“I wasn’t aware of the existence of the Food Bank, so more publicity or advertising I think it would help to let students know about it”.*	*P66, International* *P101, International*
	*Food vouchers*	*“I wasn’t earning very much and because they gave me the food vouchers, it was helpful for me to buy whatever I like and then cook by myself, and it didn’t affect my income”.*	*P85, Domestic*
***Future intervention ideas***	*Nutrition education*	*“There is not enough information, so to get informed and healthy choices, information is very, very, very, very important because let’s say there is a student who is a having a hard time and they don’t know where to go or what to do, so I think information is the most powerful tool to have healthy food choices and food security”.*	*P101, International*
	*Free fruits/food relief*	*“Maybe promoting healthy eating, but also supporting students so maybe like free fruit or something like that, that would be pretty nice”.*	*P60, International*
	*Discounts*	*“If we can tap our student ID card and if we can get like loyalty points and if we can redeem that in a restaurant or something like that, I think that would be nice”.*	*P90, International*
	*Policy*	*“Maybe a policy or like standard that shops have to follow in terms of healthy eating, kind of similar to like what we’re doing now like the plastic ban”.*	*P140, International*
	*Self-catering facilities*	*“I think it would be much more helpful if there was some area that was part of the large eating area that allowed students to do things like microwave their meals or if maybe there was like a barbecue in that giant courtyard, something where people could communally make food that they’re bringing in as opposed to having to spend that money”.*	*P130, International*
	*Food outlets*	*“I’ve been to Germany universities before and they have like their own university canteen, you know where they actually serve more healthier food, you know so instead of people going to the malls”.*	*P65, International*

#### Healthy food options on campus

Students mentioned that the most prominent opinions regarding healthy food on campus were those that contained either fruits or vegetables. Retailers predominately offering salads or sandwiches were perceived as an initial healthy option, as they contained vegetables and lower carbohydrate options. Food safety was also raised including unclean facilities, poor food preparation and presence of contaminants. When asked what they perceive to be healthy on campus (apart from mentioning food outlets), some students mentioned that nutritional composition and nutrient balances came to mind. Portion control and balanced composition of protein, carbohydrates and fibre were discussed. The consumption of unhealthy foods in moderation was raised as a concept of healthy eating at university in the absence of healthy food outlets.

#### Influences of food choices on campus

Students were asked to discuss what may influence their choices when purchasing food on campus. Key barriers to purchasing and consuming healthy foods were highlighted throughout the discussions; these were predominantly focused on price and nutritional quality. Social factors such as peer influences and culture were raised by students as an impact on their eating habits as they may be more likely to consume what their peers are eating, or foods deemed culturally safe. Students also discussed foods with nutrients that supported them with energy to focus on their studies. They discussed time and location as large influences on their choice of food whilst on campus, which demonstrates that convenience has a major role in food consumption. The most prevalent influence on student food choices was pricing as many options were too expensive for students in general. Additionally, taste preferences were highly influential in food choices and consumption for students as they wanted to eat foods with appealing taste.

#### Changes in eating habits since starting at university

To further understand how eating habits may be altered as a university student, students were asked to discuss any changes that have occurred since starting their studies. Any factors that have influenced their eating habits since becoming a student were also mentioned including time constraints and the availability of foods around campus. Due to the time commitments and class schedules many students were not able to spend as much time as they wanted cooking and preparing healthy meals. As a result, students were now more likely to purchase convenient foods deemed unhealthy such as processed fast foods. Energy requirements needed to sustain their studies since being at university were raised.

#### Impact of COVID-19 on eating habits

To gain an understanding of the impact of the COVID-19 pandemic on student eating habits and experiences with food insecurity, students were asked to share any changes that may have occurred during this time. Students discussed difficulties with accessing grocery stores during the lockdown period due to store closures and travel restrictions. They also reported issues with finding stock of healthy foods during the pandemic, leaving students to rely on unhealthy options that were available. Furthermore, the frequency of meals throughout the day may have been altered to ensure food lasted for longer. Pricing of foods during the COVID-19 pandemic was discussed by students as a large issue. As a coping mechanism, students mentioned that they were living on cheaper foods which had less nutritional value. Some students reported being more health conscious during the COVID-19 pandemic. In contrast to negative impacts of the pandemic, having time during lockdown periods allowed students to develop their capacity and gain skills with cooking and food preparation.

#### Health impacts of food insecurity

Students discussed health impacts arising from their experiences with food insecurity which comprised of physiological and psychological changes. The physiological health impacts of food insecurity were raised during the focus group and interview sessions, including weight loss and gain, loss of energy, nutrient deficiencies, headaches, and gastrointestinal issues. Students also discussed the mental impacts of food insecurity, including feelings of shame, embarrassment, guilt, and stress. This was also due to the increased prices of some healthy foods, resulting in frustration.

#### Barriers to accessing healthy foods

As students were previously asked to share the influences on their eating habits during COVID-19, and since commencing at university, an overview of the generic barriers was captured to inform future initiatives. Students mentioned that healthy options are often more expensive than unhealthy options which prevented them from eating better. Particularly, fresh fruits and vegetables as these were expensive to purchase and alternatively, students chose frozen options as they were more affordable. International students were impacted by pricing as they also had lower incomes due to restricted employment as well as those needing to provide for their family. Location of supermarkets and food outlets was a significant barrier as students would be less likely to purchase healthy foods if they had to travel a longer distance to access them. Limited options of healthy foods were also reported to influence students’ dietary behaviours. In terms of food literacy, insufficient food, and nutrition knowledge made them more susceptible to nutrition misinformation. Additionally, lack of food preparation and cooking skills were raised as a concern.

#### Views on current university support

Nutrition education within the university environment and curriculum was discussed by some students as they felt as though materials covered in tutorials or lectures provided knowledge and skills relative to healthy eating. Students discussed the food relief boxes that were available to students who were impacted by food insecurity. Although some students who experience food insecurity and accessed these boxes found them helpful, the contents were mostly canned and packaged goods which students perceived to be less healthy. Other students mentioned that they were not aware of the food relief boxes as they were not widely advertised, and the collection period was limited. Some students reported grocery vouchers provided by the university during the COVID-19 pandemic to help those who experienced food insecurity to purchase basic needs.

#### Future intervention ideas

Nutrition education emerged as a prominent theme for future initiatives as some students expressed the need to gain knowledge and awareness of healthy foods. Students suggested that statistics and detailed information on the benefits of healthy should be available online on the university’s website to encourage healthier food consumption. Additionally, students expressed that if the food outlets on campus displayed nutrition information including energy and macronutrient contents, it would allow students to know what they are consuming. Students felt free fruits available all year on campus would be beneficial, rather than having these available for shorter periods.

Students mentioned that the healthy options on campus were quite expensive, however if discounts were offered by the university, this would make purchasing these options more affordable and appealing. Reward schemes and food vouchers were also discussed where students would get a discount after purchasing from an outlet. Self-catering facilities, including food preparation amenities such as toasters and microwaves across campus were mentioned. Students felt as though having a communal area and equipment to support students with food preparation, would encourage them to bring healthy food from home whilst also allowing them to build capacity and skills regarding food preparation. Having a canteen on campus that offered ready-made healthy meals would also improve healthy eating.

Students discussed the governance of food available at the university through policy implementation. A policy on healthy eating was raised as a beneficial initiative to improve the health of students, this could also be focused on sugar regulations. Policies and monitoring relative to food safety to ensure food is safe and adheres to high standards was also raised. In addition to other interventions suggested by students, increasing healthy food outlets on campus, including a small grocery store would be helpful to purchase healthy snacks and fresh produce. Farmer’s markets on campus were discussed by students as these would encourage students to purchase fresh produce and encourage sustainability.

## Discussion

The current study aimed to comprehensively assess the university food environment and collect university students’ perspectives to inform interventions improving healthy eating and addressing food insecurity. The food environment benchmarking indicated the significant gaps within each component of the university food environment, particularly within the university’s governance system. Students reported a range of barriers to accessing healthy foods on campus.

Low scores in the food campus environment suggest that improvements are needed in the governance of the food environment through leadership, food retail policies and increased availability of affordable food options. There were no university-wide policies or frameworks identified that mentioned food or healthy eating, despite having a focus on sustainability. Previous literature highlighted that most university-wide governance documents within Australia and New Zealand predominantly focus on waste management which is consistent with findings from the food environment audit at Macquarie University [14]. Stakeholders within the university also had little to no involvement in improving the healthiness of the food environment on campus.

To improve the equity of the food environment, pricing requirements could be introduced for food retailers to ensure that a range of healthy and environmentally sustainable food options are affordably price and incentivised for consumption. Another measure to improve equity would be to implement policies that require all food retailers to display easily interpretable nutrition and sustainability labelling for all products, which would also improve capacity and education about these foods [15]. Despite the identified gaps within university governance, the university should consider building on existing efforts within community and personal development through further education and self-catering facilities to improve food preparation capacity as the results suggest that this could empower and enable students to access and consume healthier foods.

Perceptions of young people are often underrepresented and are not included in consultations for change despite them being interested in involvement, therefore, limiting the efficacy of future interventions [16]. Studies have revealed that engagement with young people is essential for transformation and meaningful change due to their influence and insight into contemporary issues[16, 17]. Many studies have previously assessed this topic with a singular approach, including an audit without student consultation or qualitative consultations without a comprehensive assessment of the environment [18–22]. The current study utilised a combined approach to holistically assess the university food environment. The assessment of multiple components of the food environment allows for a comprehensive review to determine key facilitators and barriers, including individual and physical factors [23].

During the qualitative component of the study, students discussed the wider influences of their food choices whilst on campus, including socio-cultural factors such as peer influence, nutritional quality of foods, time and location, price, and taste. These findings were consistent with previous research that found university students to consume unhealthier foods knowingly due to the convenience, lower price point and peer influence [24]. Additionally, students reported that taste preferences had a large impact on their food choices whilst on campus as they were less likely to consume healthier foods which were less appealing. Individual influences including personal beliefs and preferences, self-discipline in conjunction with external influences from their peers and environment are known to have a significant effect on a student’s dietary behaviours, which align to the discussions from this qualitative component of the study[25–27].

Since starting at university, many participants, particularly international students, discussed major changes to their eating habits, including the shift from preparing meals at home to purchasing more processed meals. These findings are consistent with prior studies as many students have shifted towards a westernised, processed diet since commencing at university, particularly displaced students who have experienced disruptions to their studies due to a crisis or major change in their lives[28, 29].

Students also discussed the decreased frequency of meals due to class schedules and study commitments throughout the day, which aligned with prior literature suggesting that dietary intake of first year university students had rapidly declined in comparison to before commencing their studies [30]. Despite this, many students felt they had still met their nutrient requirements as some often ate larger meals or binged on foods, at single points throughout the day which is commonly seen in students [30]. Students also reported that they needed to consume foods that would provide them with energy to sustain their studies as it would assist them in achieving better academic outcomes. Healthier diets, including adequate fruits and vegetables intake were identified to be correlated with improved focus, memory, mood and academic outputs[31–33].

When asked to discuss their experiences with healthy eating and food security throughout the COVID-19 pandemic, participants mentioned that access to food and pricing were the main barriers. Government lockdowns impacted the ability to access supermarkets and participants were restricted to their local areas which may not have had healthy options readily available due to supply issues[34, 35]. During these times, many participants mentioned that they consumed less foods or consumed unhealthier options due to pricing to balance their finances in the absence of a stable income [35].

Apart from discussing negative impacts, participants also discussed the positive changes in their dietary patterns and capacity due to increased time to build and develop food preparation skills. Due to restaurant closures, many students opted to spend time cooking their own healthy meals which they did not report doing prior to the pandemic. These results are similar to a study conducted in Brazil, revealing that 70% of participants had increased their cooking skills during the pandemic, using mostly fresh ingredients [36]. These findings were also consistent within an Australian context as home cooking and experimentation occurred during the pandemic, leading to improved food literacy [37].

The most common themes related to health impacts were physiological and metabolic changes, including weight gain and loss, headaches, increased stress and body image concerns. Whilst many studies have focused on the psychological impacts of being food insecure, including depression and anxiety, these often lead to physical outcomes which can have a detrimental impact on a young person’s health[38–40]. Students who consume an insufficient quantity of nutritionally adequate foods, may experience malnutrition, nutrient deficiencies, and energy loss [40, 41]. Students who consume processed, fast foods as a coping mechanism may be at risk of obesity and other chronic diseases, including type 2 diabetes and cardiovascular disease[38, 39].

The key initiatives discussed to improve food security and healthy eating were university nutrition education, food relief boxes and vouchers, free fruits on campus, increased healthy food outlets, discount and reward schemes, food preparation facilities, and improved food governance. Food relief boxes were beneficial as they assisted students with immediate relief to ensure that they did not experience hunger, however, they felt as though the contents were not supportive of healthy lifestyles due to the absence of healthy, fresh foods, these discussions were consistent with prior findings[42–44].

In contrast to this, students discussed discount schemes and food vouchers for supermarkets to be beneficial in promoting healthy eating as they were able to purchase fresh produce whilst encouraging food preparation skills. Self-catering facilities on campus including microwaves, toasters, and communal spaces would also promote healthy cooking [45]. Free fruits and outlets offering fruits and vegetables on campus were identified as an ideal option to encourage healthy snacking, especially during university events where unhealthy food would otherwise be promoted[41, 42, 46].

Additionally, participants also raised that governance of the food environment may be required to ensure food safety standards are withheld alongside regulations of unhealthy foods. Studies have revealed that universities in other countries have implemented legislation regarding unhealthy food and beverage taxations, marketing restrictions, subsidies on healthy options, these can assist in preventing non-communicable diseases [47, 48].

### Strengths & limitations

This study had various strengths which allowed for an accurate investigation and representation of students’ experiences with healthy eating and food insecurity. Using food environment analysis was a strength as data was collected by three assessors individually which was then cross-referenced and validated by an external, independent experienced team of researchers from another university for validation and to decrease reporting biases. Both the focus groups and semi-structured interviews were exploratory and allowed students to discuss their experiences in detail with limited guidance. Whilst the general topics were guided by the facilitator, there was flexibility in the discussion and this approach allowed students to share their views, attitudes, and ideas without any restrictions. By dividing students into two groups based on their student status, it prevented any contentious discussions due to different experiences and vulnerability of these populations throughout the pandemic due to differences in government support.

This study had some limitations within both components. The Uni-Food tool does not consider the proximity of the university to shopping centres, the presence of a nutrition and dietetics department, and food outlets that serve alcohol on campus. These factors may influence the score and affect the healthiness of the university food environment. The use of purposive sample to recruit students identified as food insecure for focus groups and semi-structured interviews was another study limitation, which may not reflect other students’ experience who are deemed to be food secure. Moreover, changes in eating habits during the pandemic and since commencing university were self-reported narratives which may present reporting and recall biases.

## Conclusions

This study highlights the need to comprehensively measure the food environment and consult students to identify the key barriers and enablers of healthy eating and food insecurity. Availability, pricing, location, taste preferences, knowledge, convenience, and nutritional quality of foods were influential factors to improve healthy eating on campus. The benefits of short-term assistance in accessing foods provided by the university were deemed to be limited. The direction of future interventions on campus could build on the existing efforts to ensure that relief options are sustainable and supportive of students’ healthy lifestyles through discounts, food preparation facilities. policies, and education. Additionally, the university should conduct further consultations with a wider sample of university students and stakeholders to determine the feasibility of these potential interventions to ensure that they will be effective in improving healthy eating and reducing food insecurity for students on campus.

### Electronic supplementary material

Below is the link to the electronic supplementary material.

Supplementary Material 1

## Data Availability

The data presented in this study are not publicly available but are available from the corresponding author on reasonable request.

## Abbreviations

COVID-19: Coronavirus Disease 2019
PICF: Patient Information and Consent Form
