# Supplementary material for: Benchmarking the university campus food environment and exploring student perspectives about food insecurity and healthy eating: a case study from Australia
Source: BMC Public Health. 2024 May 6;24:1245. doi: 10.1186/s12889-024-18664-x (PMC11075336; doi:10.1186/s12889-024-18664-x)
Supplement: Supplementary file 1 — Supplementary Material 1 [file 12889_2024_18664_MOESM1_ESM.docx]

Supplementary Materials

Supplemental Material S1: Student Focus Group and Interview Guide

This guide was used to conduct qualitative data collection of student experiences with food insecurity and healthy eating on campus.

*Topic one: Healthy food on campus*

1. Thinking of healthy food on campus, what comes to your mind?

- 1. 2. When you consider healthy food options at this university, which factors have the most influence on your food choice? a. Think about price, availability, variety, time, taste preferences, knowledge, skills and social influences
  2. 3. Have any changes to your eating habits happened since you started university? If so, what were they?

*Topic two: Food insecurity and its impacts*

You may or may not have experienced changes in eating habits during the pandemic or even otherwise. Our previous research suggests that some students have experienced food insecurity during the pandemic and this finding has been shown in other Universities as well.

**Food insecurity** is a situation when you lack regular access to obtain enough food due to lack of money, unavailability, distance to purchase food.

In some cases, you may have experienced:

- Lack of money to purchase healthy food.
- You cannot obtain healthy food because it’s not available or it’s required to travel a long distance.
- Skipping meals or running out of food occasionally
- Gone on entire day without eating at times during the year.

1. Please discuss your personal circumstances with food and how you have ensured you get enough?
   1. Think about access to enough quantity of foods or nutritional quality of foods.
   2. Think about the priority: size, quantity, healthy and price.
2. How has the COVID-19 pandemic impacted your ability to assess enough food?
3. Comparison: before and after the outbreak
4. What do you think are the main barriers and enablers to accessing food?
5. Think about living arrangement situations (e.g., living with family or alone), job, financial situation.
6. Tell me about how your situation with food may have impacted your health?
7. How often have you felt stressed or anxious due to not being able afford or access food?
8. Has this made you feel ashamed or embarrassed?

*Topic three: University support*

8. When you think of the general conditions at Macquarie University, to what extent do they support you in following a healthy diet?

9. How do you think to improve the availability and affordability of healthy food on campus?

- 1. 10. In regard to food insecurity, have you ever accessed support services within the university?
  2. a. To what extent it is helpful? (Example: receiving a hamper from Foodbank during the COVID-19 lockdown?)

*Topic four: Solutions*

11. Tell me about how you feel you could be further supported by the university to improve healthy eating and reduce food insecurity on campus?

One of the options we are thinking about is establishing a food coop at the University. Food co-operative is where fresh fruit and vegetables are purchased in bulk every week and distributed at wholesale prices among students who are interested in joining.

One of the food coops is the Box Divvy Organisation. the Box Divvy is a unique group of food coops that aims to provide minimally packaged and minimally processed, affordable, wholesome, organic food to its members. They offer organic food both fresh produce and grocery items, support local farmers, less plastic, less waste and supply high-quality products at reasonable prices. [playing short video link: https://youtu.be/bvrP6nf13aM]

12. What do you know about food cooperatives?

- 1. 13. What do you think about food cooperatives to improve healthy eating and reduce food insecurity on campus?
  2. a. Think about feasibility, long-term
  3. b. Would you like to be involved in helping us to set up a food coop here at the university?
  4. c. Would you use a food coop if it was available?
  5. 14. Are there any other thoughts or comments you would like to share?

Supplemental Figure S2: Key themes and sub-themes of student experiences with food insecurity and healthy eating on campus collected during focus groups and interviews.

**
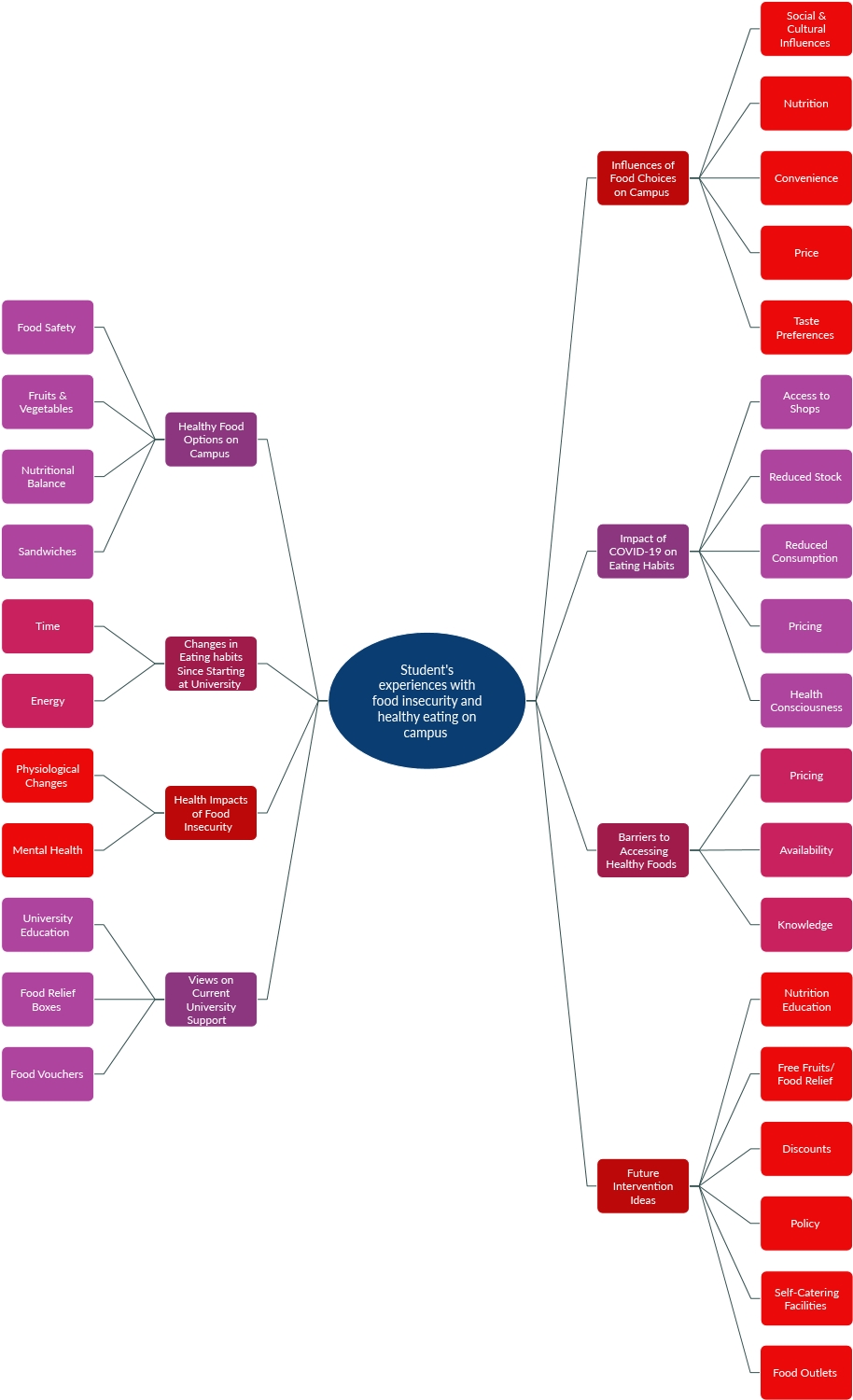
**
